# Dr. Herbert Gordon Jones

**Published:** 1917-08

**Authors:** 


**Dr. Herbert Gordon Jones,** N. Y. University 1881, died at his home in Utica, July 5, aged 59. He was Ophthalmic Surgeon, Utica Masonic Home, Attending Surgeon to St. Luke's Hospital and Coroner of Oneida Co. He was a past president of the County Society.

